# The splicing landscape is globally reprogrammed during male meiosis

**DOI:** 10.1093/nar/gkt811

**Published:** 2013-09-12

**Authors:** Ralf Schmid, Sushma Nagaraja Grellscheid, Ingrid Ehrmann, Caroline Dalgliesh, Marina Danilenko, Maria Paola Paronetto, Simona Pedrotti, David Grellscheid, Richard J. Dixon, Claudio Sette, Ian C. Eperon, David J. Elliott

**Affiliations:** ^1^Department of Biochemistry, University of Leicester, Leicester, LE1 9HN, UK, ^2^Institute of Genetic Medicine, Newcastle University, Newcastle upon Tyne, NE1 3BZ, UK, ^3^School of Biological and Biomedical Sciences, Durham University, Durham, DH1 3LE, UK, ^4^Department of Health Sciences, University of 00135 Rome ‘Foro Italico’, Rome, Italy, ^5^Laboratories of Neuroembryology and of Cellular and Molecular Neurobiology, Fondazione Santa Lucia IRCCS, 00143 Rome, Italy, ^6^Department of Public Health and Cell Biology, University of Rome Tor Vergata, 00133 Rome, Italy, ^7^Institute of Particle Physics Phenomenology, Durham University, Durham, DH1 3LE, UK and ^8^Life Technologies Ltd., Paisley PA4 9RF, UK

## Abstract

Meiosis requires conserved transcriptional changes, but it is not known whether there is a corresponding set of RNA splicing switches. Here, we used RNAseq of mouse testis to identify changes associated with the progression from mitotic spermatogonia to meiotic spermatocytes. We identified ∼150 splicing switches, most of which affect conserved protein-coding exons. The expression of many key splicing regulators changed in the course of meiosis, including downregulation of polypyrimidine tract binding protein (PTBP1) and heterogeneous nuclear RNP A1, and upregulation of nPTB, Tra2β, muscleblind, CELF proteins, Sam68 and T-STAR. The sequences near the regulated exons were significantly enriched in target sites for PTB, Tra2β and STAR proteins. Reporter minigene experiments investigating representative exons in transfected cells showed that PTB binding sites were critical for splicing of a cassette exon in the *Ralgps2* mRNA and a shift in alternative 5′ splice site usage in the *Bptf* mRNA. We speculate that nPTB might functionally replace PTBP1 during meiosis for some target exons, with changes in the expression of other splicing factors helping to establish meiotic splicing patterns. Our data suggest that there are substantial changes in the determinants and patterns of alternative splicing in the mitotic-to-meiotic transition of the germ cell cycle.

## INTRODUCTION

Most mammalian protein-coding genes are comprised of multiple exons and introns. Introns are generally much longer than exons and are removed from the initial transcript by pre-mRNA splicing. In ∼90% of genes, multiple mRNA isoforms are produced as a result of either the existence of multiple splicing pathways (alternative splicing) or the use of different promoters or termination sites (1–3). Many alternative exons have been found (4) through sequencing of full-length mRNAs and expressed sequence tags. Each human protein coding gene produces an average of 11 mRNA isoforms through alternative splicing, and recent estimates suggest there are >82 000 transcriptional initiation sites and 128 000 alternative polyadenylation sites for ∼21 000 human protein coding genes (5). As the use of many splice sites and alternative promoters or polyadenylation sites is regulated in response to extracellular cues or during development, alternative mRNA isoforms can determine the functions of a gene in different circumstances.

Because splicing amplifies the functional content of the genome, there is currently great interest in how both RNA splicing regulators and mRNA isoforms are modulated in development (6). Extensive splicing switches have been found in the heart, the immune system and brain (7–11), and some human diseases such as myotonic dystrophy are caused by defects in developmental splicing (12). Spermatogenesis is one of the most radical pathways of development still maintained in adult animals. Spermatogenesis involves alterations in both chromosome number and cell morphology to convert a diploid stem cell in which chromatin is packaged with histones into a motile haploid cell with a compact nucleus containing chromatin packaged with protamines. Exon-specific microarrays have detected more alternative splicing in the whole adult testis than in any other tissue except the brain (13), although at what stage in spermatogenesis this splicing regulation originates is not known. Perhaps the most important question concerning changes in alternative splicing patterns during male germ cell development is whether it is connected to meiosis. Unlike cells in mitosis, in which transcription is turned off, meiotic cells are highly transcriptionally active (14). In the single-celled yeast *Saccharomyces cerevisiae*, meiosis is the only stage in the lifecycle to include alternative splicing. In fact, the timing of the gene expression changes that drive meiotic progression in yeast is determined by a hierarchy of meiotic splicing events (12,15).

**Figure 1.**
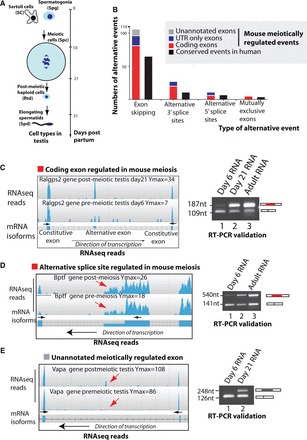
High frequency switches in mRNA isoforms take place between the mouse pre-meiotic and meiotic testis transcriptomes. (**A**) Cartoon showing major cell types in the mouse testis, and the timing of their appearance in the postnatal mouse. (**B**) Summary of the patterns of alternative splicing found by comparative RNAseq analysis to change between 6 and 21 dpp in the mouse testis transcriptome (this table summarizes information in Supplementary File S1). (**C**) Example of a meiotically regulated alternative cassette exon in the *Ralgps2* gene. (**D**) Example of a meiotically regulated alternative splice site in the *Bptf* gene. (**E**) Example of a previously unannotated exon discovered in the mouse *Vapa4* gene, which is regulated during meiosis. In parts (C and D), the patterns of exon inclusion were monitored during meiosis by direct visualization of RNAseq reads on the Savant genome browser (16) indicating the maximum peak (Ymax) of reads at 6 and 21 dpp (left hand panels for each gene), and by RT-PCR using primers in flanking exons (Supplementary Table S1) followed by agarose gel electrophoresis (right hand panels for each gene).

Adult male germ cell development takes around 30 days in the testis (Figure 1A). Male mice are not born with a fully developed male germ cell development pathway, and instead the first wave of spermatogenesis is initiated synchronously after birth (17). The testes of newborn mice contain germ cells arrested at the G_0_ and G_1_ stages of the cell cycle. By 6 days post partum (dpp), these germ cells have differentiated into spermatogonia, a cell population that includes stem cells. At ∼10 dpp, some spermatogonia differentiate into meiotic spermatocytes. Meiosis commences ∼12 dpp. The first meiotic division (meiosis I) is complete by 21 dpp, after which there is a rapid second cell division (meiosis II) followed by progressive differentiation of the haploid cells into round spermatids, elongating spermatids and finally mature sperm (this haploid differentiation process is called spermiogenesis). Distinct programmes of transcriptional changes take place over animal meiosis, which are critical for driving different molecular events such as the expression of genes encoding synaptonemal proteins like Sycp1 and the recombinase Spo11 (18–20). It might be anticipated that important splicing patterns represented only in testis would be linked likewise to meiosis. In the very last stages of male germ cell development, nuclear DNA condenses during the differentiation into elongating spermatids, limiting transcriptional activity. At this time, translational control of pre-existing mRNAs transcribed earlier in meiosis plays a key role in gene expression (21).

Much of the alternative splicing that has been detected in whole human testis by exon-specific microarrays is not conserved in mice leads to the introduction of premature stop codons, and it occurs at individually low frequencies (13). These characteristics suggest that some alternative splicing may represent ‘noise’ (22), arising from a lower stringency of splicing control in the testis. However, there are changes in the levels of expression of a number of important RNA splicing regulators during spermatogenesis, including heterogeneous nuclear RNP (hnRNP) proteins (hnRNPA1, hnRNPG and PTB) (23–26), SR-like proteins (Tra2β) (27) and STAR proteins (Sam68 and T-STAR) (28,29). In this study, we have set out to identify whether there are high frequency changes in global splicing patterns that could affect protein isoform production over meiosis and to comprehensively monitor the expression of RNA splicing regulator proteins over this timeframe. Interestingly, PTB is essential for male germ cell development in fruitflies (30,31). We speculate that although polypyrimidine tract binding protein (PTBP1) is downregulated in meiosis, nPTB might functionally replace it and thus ensure correct regulation of PTB-dependent splicing events during germ cell differentiation. Moreover, other RNA splicing regulators also change in abundance during germ cell differentiation, suggesting that they contribute to the meiotic patterns of splicing that we observe.

## MATERIALS AND METHODS

### RNA sequencing

Sequencing reactions were done on pooled RNA samples from 6 and 21 dpp mouse testis by Source Bioscience, Nottingham, UK. Four lanes of the flowcell were used for the sequencing of the 6 and 21 dpp samples on the Genome Analyzer II. The Genome Analyzer (GA) was run for 38 cycles. The images from the GA were analyzed with the GA pipeline software (v1.3, Illumina software) on cycles 1–38 to undertake image analysis, base calling and sequence alignment to the reference genome. In all, 5 345 040 and 5 561 352 quality filtered reads were obtained for the 6 dpp sample, and 7 629 529 and 7 610 503 were obtained for the 21 dpp sample. The mouse NCBI Build 37.1 (mm9) was used as a reference genome used for the read alignment. Sequences were aligned with the ELAND software (‘ELAND_rna’ option), which resulted in 3 922 430, 4 085 374, 5 515 372 and 5 504 414 aligned reads for the 6 and 21 dpp samples, respectively. The aligned reads were used as input for the Illumina CASAVA program (v1.0) to count the sequence reads that align to genes, exons and splice junctions of the reference genome. The mouse reference feature files were supplied by Illumina and were derived from the mouse NCBI Build 37.1. The raw counts of sequences aligning to features (gene, exons and splice junctions) were normalized by CASAVA by dividing the raw count by the length of the relevant feature.

The read counts per gene were used as input for DEGseq (32) and DESeq (33) to identify differentially expressed genes. Both tools are available via the statistics package R and Bioconductor. DEGseq and DESeq use different statistical approaches (Poisson distribution, negative binomial distribution) to estimate probabilities for differential gene expression. A *P* ≤ 0.001 and a 2-fold change (normalized) in expression levels were used as cut-off criteria. Using these cut-offs, DESeq identified 5835 genes as differentially expressed, whereas DEGseq found 6362 differentially expressed genes. The common set of 5296 genes was taken as comprising the differentially expressed genes for further analysis. The resulting list was read into the GOseq (34) Bioconductor/R-package to identify GO terms that are over- or under-represented. GOseq corrects for length bias in the detection of differential expression in RNAseq. The relationship between gene expression for every gene in our data set before and after meiosis (6 and 21 dpp, respectively) was represented using scatter plots prepared using an in-house Python script. Read counts per gene were used as an input and were derived from CASAVA.

The MISO pipeline (35) was used to identify differential alternative splicing across the 6 and 21 dpp samples. Briefly, MISO requires a library file of annotated alternative events and alignment files for the two stages as input. The mm9 alternative event annotation file (36) as provided with the MISO software was used as a library file. For the events defined in the library file, MISO measures for differential expression using Bayesian inference. To generate MISO-compatible alignment files, the quality filtered reads for the two stages were re-aligned against the mm9 mouse reference genome with Tophat (37), using the Illumina mm9 genome feature file to improve the detection of splicing junctions. The Fastmiso version of the MISO package was run with default settings. A combination of different cut-offs and filters was tested in the analysis of the MISO output, culminating in the use of a Bayes factor of 10 as cut-off value to detect differential alternative splice events. RNAseq reads were visualized on the mouse genome using the Savant genome browser (16).

### Analysis of enriched sequences associated with meiotic splicing regulation

K-mer analysis was carried out using custom scripts as described previously and the total set of cassette exons predicted as meiosis-regulated by MISO (35,36). We chose a bayes factor value of >10 from the MISO results as a cut-off to define exons that were alternatively spliced in meiosis, and exons over 500 bases (9 exons) were removed, to yield a total of 251 exons that are alternatively spliced in meiosis. The background data set was defined as the set of exons smaller than 500 bp and with a bayes factor value below 0.1, indicating that although they were expressed in our data set, they were not alternatively spliced (276 exons). We analysed alternative exons and 250 bases of flanking introns with corresponding background data sets. Activated (159 exons) and repressed (92 exons) data sets predicted by MISO were analysed separately with the same background data set to identify enriched 5-mers that were over-represented in meiotic regulated exons. The 5-mer counts were normalized to the corresponding data set size (frequency) as well against the background. K-mers were ranked in order of the highest difference to the background, and significance was measured using a *t*-test. The complete k-mer list with counts for all possible 5-mers is presented in the Supplementary Data. Potential binding sites for PTB were analysed as described (38), with the spacing relaxed to YCUN_(1–6)_CUN_(1–8)_YCU, where N is any nucleotide. For analysis at lower stringency, a match was only required at 7 of the 8 nt specified in YCUN_(1–6)_CUN_(3–8)_YCU.

### Amplification of different mRNA isoforms

Candidate meiotically regulated splice isoforms were characterized by RT-PCR using the primer sequences given in Supplementary File S1, followed by either agarose gel electrophoresis or capillary gel electrophoresis for quantitation. Percentage Splicing Inclusion (PSI) values were calculated as the concentration of isoform including alternative event/(concentration of isoform including alternative event + concentration of isoform excluding alternative event) × 100. Heat maps were drawn using http://www.hiv.lanl.gov/content/sequence/HEATMAP/heatmap.html.

### Cell isolation

Spermatogonia were obtained from 7 dpp CD1 mice (Charles River, Italy) as previously described (39); Sertoli cells were prepared from 7 and 17 dpp CD1 mice as previously described (40). Testes from 28–30 dpp CD1 mice were used to obtain pachytene spermatocytes and round spermatids by elutriation (41). Purified germ cells were collected, washed with phosphate-buffered saline (PBS) and used for RNA and protein extraction. To analyse the timing of splicing events in meiosis, RNA samples were analysed from 13 dpp testis (latest stage: early meiosis), 16 dpp testis (latest stage: early pachytene), 18 dpp (latest stage: late pachytene and meiotic divisions) and day 21 (meiosis complete).

### RNA and protein extraction

*Purification of RNA from tissues*. 

Total RNA from whole postpartum testes or adult mouse tissues was isolated using TRIZOL (Invitrogen). Poly A^+^ RNA was purified using a Dynabeads mRNA purification kit (Invitrogen). Parallel samples were fixed using Bouin’s and mounted in paraffin wax, followed by H&E staining using standard procedures as previously described (42). Total RNA from isolated germ cells or Sertoli cells was prepared using TRIZOL (Invitrogen) according to the manufacturer’s instructions. DNase digestion was performed using RQ1 RNase free DNase (Promega) at 37°C for 20 min. One microgram of RNA was used for RT-PCR with the Superscript III reverse transcriptase (Invitrogen) according to manufacturer’s instructions. A total of 5% of the RT reaction was used as template for the PCR reaction. Oligonucleotides used as PCR primers are listed in the Supplementary File S1.

For protein extraction, cells were washed in ice-cold PBS, homogenized and lysed in lysis buffer (50 mM Hepes (pH 7.4), 150 mM NaCl, 15 mM MgCl2, 15 mM EGTA, 1% Triton X-100, 10% glycerol, 20 mM β-glycerophosphate, 1 mM DTT, 0.5 μM Na_3_VO_4_) and protease inhibitors (Sigma Aldrich). After 10 min on ice, cell lysates were centrifuged at 10 000 *g* for 10 min at 4°C. Cell extracts were diluted in Laemmli sample buffer and boiled for 5 min.

### Western blot analysis

Proteins were separated on 10% SDS–polyacrylamide gels and transferred to polyvinylidene fluoride Immobilon-P membranes (GE-Healthcare) using a wet blotting apparatus (Bio-Rad). Membranes were saturated with 5% BSA at room temperature and incubated with the following primary antibodies (1:1000 dilution) at 4°C overnight: α-nPTB; mouse α-hnRNP A1, α-hnRNP A2/B1, α- hnRNP C1/C2, α-SC35 (Sigma Aldrich); mouse α- hnRNP F/H (Abcam); rabbit α-SRp55, α-SRp20, α-SRp40, α-ERK2 and goat α-hnRNP I (Santa Cruz Biotechnology); mouse α-ASF/SF2 (US Biological). Secondary anti-mouse, anti-goat or anti-rabbit IgGs conjugated to horseradish peroxidase (Amersham) were incubated with the membranes for 1 h at room temperature at a 1:10000 dilution. Immunostained bands were detected by a chemiluminescent method (Santa Cruz Biotechnology).

### Minigene analysis

Minigenes were cloned into pXJ41 using the primers in Supplementary File S1, and mutagenesis was carried out by overlap PCR as previously described (27).

## RESULTS

### High frequency switches in mRNA isoforms take place between the mouse pre-meiotic and meiotic testis transcriptomes

Previous transcriptome-wide analyses of gene expression changes in meiosis have detected only a single expression signal per gene and so have been unable to detect changes in mRNA isoforms (18–20). To comprehensively profile gene expression changes taking place during meiosis, we initially took advantage of the synchronous onset of meiosis in the testes of new-born mouse to separate gene expression changes in meiosis from those associated with the later processes of morphological differentiation (17). Testes were dissected from mice before (6 dpp) and at the end of meiosis (21 dpp) (Figure 1A). PolyA+ RNA was isolated from testes at both ages, and then analysed by deep sequencing (RNAseq).

We analysed this RNAseq data (35) to identify a pool of alternative splicing changes that occur between the 6 and 21 dpp testis transcriptomes. From the total alternative events predicted by the MISO programme, we selected 104 exon skipping events, 11 alternative 5′ splice sites, 28 alternative 3′ splice sites and 5 mutually exclusive exons by visual inspection (Figure 1B and Supplementary File S2). We experimentally confirmed 15 of 20 tested events from these regulated events using RT-PCR analysis, a validation rate of 75% (e.g. Figure 1C–E right panels and Supplementary File S2). Although we detected alternative splicing of some 5′ UTR and poison exons, most detected alternative splicing events regulated in meiosis introduced exon sequences that comprised integer multiples of three nucleotides and were protein coding (Figure 1B). Such events included meiotic inclusion of a cassette exon within the *Ralgps2* mRNA, which encodes a ras-specific guanine nucleotide-releasing factor, and an alternative 5′ splice site in the *Bptf* mRNA, which encodes a bromodomain PHD transcription factor (Figure 1C and D, respectively).

Several of the exons regulated during postnatal mouse testis development were also annotated as alternative events in the human genome, including *Ralgps2* and *Bptf* (43) (Figure 1B). RNAseq analysis also predicted meiotic splicing regulation of a number of exons currently unannotated on the mouse genome browser, including one in the mouse *Vapa4* mRNA, which we confirmed experimentally (Figure 3D). Some of these currently unannotated exons (including that in *Vapa4*) mapped to regions of chromosome conservation between species and were already annotated as either alternative or constitutive exons in the human genome (Supplementary File S2).

### Regulated splicing events take place between spermatogonia and spermatocytes

**Figure 2.**
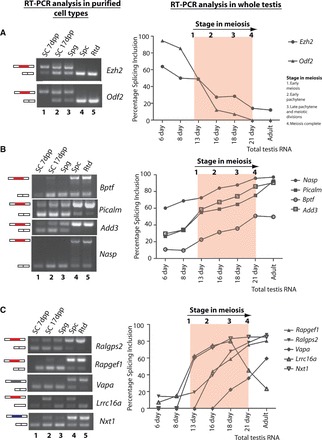
Splicing events that change between spermatogonia and spermatocytes. (**A**) Cassette exons in the *Ezh2* and *Odf2* genes are repressed during meiosis. (**B**) An downstream 5′ site in the *Bptf* gene and cassette exons in the *Picalm*, *Add3* and *Nasp* genes are activated during meiosis. (**C**) Cassette exons in the *Ralgps2*, *Rapgef1*, *Vapa4*, *Lrrc16a* and *Nxt1* genes are activated during meiosis. Left hand panels: Levels of the different mRNA isoforms were detected by RT-PCR in RNA from purified cell types, using primers in flanking exons (Supplementary Table S1) followed by agarose gel electrophoresis. The different kinds of splicing event are annotated as in Figure 1, with protein coding events in red, UTR exons in blue and previously unannotated events in grey. Right hand panels: levels of PSI in the testis at different days after birth (the first wave of meiosis is highlighted in red). SC, sertoli cells (isolated at 7 and 17 dpp); Spg, spermatogonia; Spc, primary spermatocytes; Rtd, round spermatids.

We confirmed the cell type-specificity of the observed splicing changes using RT-PCR analysis of RNA purified from cell types in the adult testis (Figure 2A–C, left hand panels). In 13 of 14 alternative splices tested in this way, splicing changed between spermatogonia and spermatocytes, confirming their splicing was regulated during meiosis (Figure 2 and Supplementary File S2). Confirmed meiotic splicing changes included activation of the *Ralgps2* cassette exon and the downstream splice site in *Bptf* (Figure 2B and C, left hand panels). We also observed a switch to complete repression of the cassette exons in the *Odf2* and *Ezh2* mRNA (Figure 2A, left hand panel).

Analysis of purified cell types indicated that for some exons, splicing regulation also occurs in Sertoli cells. Generally, developmental splicing switches in Sertoli cells occurred at a lower frequency than those observed in meiotic cells. An exception was for alternative splicing regulation of the *Lrrc16a* mRNA, which encodes a leucine-rich protein (Figure 2C, left hand panel). *Lrrc16a* showed a similar switch in mRNA splice isoforms between spermatogonia and spermatocytes and between 7 and 17 dpp Sertoli cells. Although most splicing isoform switches established in meiosis were maintained in round spermatids, *Lrrc16a* again was an exception. *Lrrc16a* mainly produced the exon-skipped mRNA splice isoform in spermatogonia and spermatids, and the exon-included isoform in meiosis.

Although the aforementioned experiments analysed the profile of mRNA splice isoform switches, which take place between pre-meiotic and meiotic cells, meiosis itself takes place over 12 days in the mouse. To monitor more precisely the timing of splicing regulation during mouse meiosis, we analysed splicing patterns of this same panel of exons during the first wave of spermatogenesis (Figure 2A–C, right hand panels). Meiotic switches in many mRNA isoforms (including *Odf2*, *Ezh2*, *Add3*) started early in meiosis (by day 13, which is 1 day after meiosis initiates in male mice). Later events included *Rapgef1* (13 dpp) and *Vapa4* (16 dpp) (Figure 2C, right hand panel). Consistent with the results from purified cell types, the splicing pattern of *Lrrca16a* switched back to mainly the exon-skipped form in adult testis.

### Most meiotically enriched splice isoforms are testis-enriched rather than meiosis-specific

The aforementioned analyses indicate the existence of a pool of meiotic splicing switches. These events might occur only in the testis, during and after meiosis, or they might occur elsewhere in the body in response to different regulatory signals. To test this, we purified RNA from other mouse tissues and analysed splicing patterns using RT-PCR.

**Figure 3.**
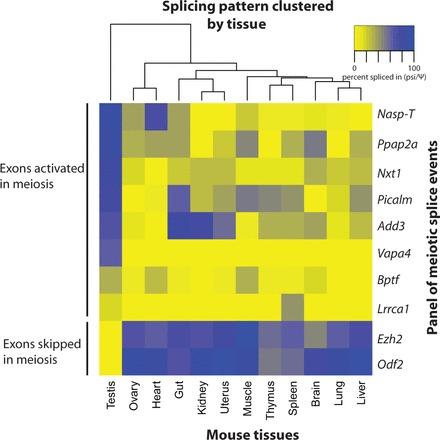
Most meiotically enriched splice isoforms are testis-enriched rather than meiosis-specific. Heat map showing PSI levels of each of the meiotically regulated exons in different mouse tissues. PSI levels are clustered according to tissue (horizontal axis) and splicing pattern (vertical axis). Patterns of expression are organized so that the exons showing the highest levels of inclusion in the testis are seen at the top of the vertical axis. PSI levels were measured using RT-PCR analysis using RNA purified from different mouse tissues, using the primers in Supplementary File S1.

When splicing inclusion levels were analysed in different tissues of the adult mouse (horizontal clustering in Figure 3), the testis formed an outlier group for both meiosis-activated and meiosis-repressed exons, indicating that meiosis-regulated splicing events are differentially regulated in the mouse testis compared with other tissues. Complete exclusion of both the *Odf2* and *Ezh2* meiosis-repressed exons was only found in the testis, and splicing inclusion of the cassette exon in *Vapa4* was only observed in the testis. However, most meiotically regulated exons in mouse testis were included to some extent in other mouse tissues as well. For example, the *Nasp-T* exon is spliced into mRNAs in the mouse heart, and the *Add3* cassette exon is included at high levels in the mouse gut and kidney.

We also used the RNAseq data to compare overall gene expression patterns of genes with activated and repressed cassette exons between the 6 and 21 dpp testis. Many genes with meiotically regulated cassette exons also increased in overall gene expression between the 6 and 21 dpp testis transcriptomes (Supplementary Figure S1A and B and Supplementary File S3). For the *Nasp* and *Odf2* genes (which have known important roles in germ cell development, see ‘Discussion’ section), we also found that that distinct transcriptional initiation sites were used in meiosis (indicated by red arrows in Supplementary Figure S1C and D). To validate these gene expression patterns inferred from the RNAseq data set, we analysed the patterns of expression of genes already known to be regulated over meiosis (Supplementary Figure S2 and Supplementary File S4). Genes known to be involved in the mouse meiotic gene expression programmes (18) were more highly expressed in the 21 dpp testis, including *Ccna1*, *Aurkc*, *Spdy1*, *Acrbp*, *Adam2*, *Adam18*, *Pla2g6*, *Ribc2*, *Tcfl5*, *Ppp3r2*, *Smcp* and *Spag6.* In contrast, known members of the core mitotic programme (*Gata4*, *Dmrt1*, *Osr2*, *Pcdh18* and *Abca1*) were more highly expressed in the 6 dpp testis than the 21 dpp testis (18).

### Comprehensive analysis of splicing factor gene expression show global changes in the meiotic splicing regulator landscape

**Figure 4.**
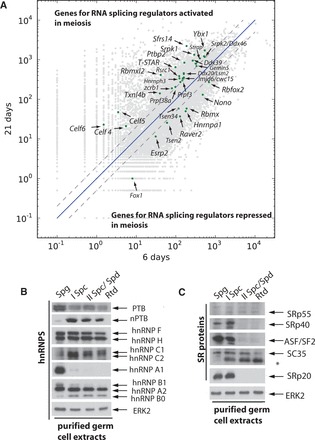
Comprehensive analysis of splicing factor gene expression showing changes in the meiotic splicing regulator landscape. (**A**) Scatterplot showing expression levels of genes encoding known RNA splicing regulators (shown as green dots) that change expression >2-fold (broken diagonal line) between the 6 and 21 dpp testis transcriptomes. A full alphabetical list showing changes in RNA splicing factor expression between the 6 and 21 dpp testis of all known RNA splicing regulators is given in Supplementary File S3. (**B**) Western blot analysis of hnRNP proteins in extracts made from cell types purified from the adult mouse testis. Spg, spermatogonia; I Spc, primary spermatocytes; II Spc/Spd, secondary spermatocytes and elongated spermatids; Rtd, round spermatids. (**C**) Western blot analysis of SR proteins in extracts made from cell types purified from the adult testis. The asterisk indicates a non-specific band detected by the α-SC35 antibody.

RNA splicing regulation is under combinatorial control, with an important role for RNA-binding protein expression (44,45). To comprehensively analyse changes in the splicing landscape in meiosis, we monitored the expression of all known RNA splicing regulators between the 6 and 21 dpp testis (Figure 4, Supplementary Files S5 and S6). Identified changes in expression included the 2-fold downregulation of *Ptbp1* (encoding PTBP1 protein), whereas *Ptbp2* (encoding nPTB protein) was upregulated 5-fold, with a similar isoform switch at the protein level (Figure 4A and B). Interestingly, transcription of *Raver2*, which encodes a protein that interacts with PTB (46), was also significantly downregulated in the 21 dpp testis transcriptome, consistent with a coordinate modulation of PTB activity in meiotic cells.

Amongst the other genes encoding hnRNP proteins, we observed an isoform switch between expression of the X chromosome-encoded *Rbmx* gene, before meiosis, to the autosomal retrogene *Rbmxl2* during and after meiosis (Figure 4A) (26,47). RNAseq analysis also detected a decrease in expression of *Hnrnpa1* mRNA between 6 and 21 dpp. HnRNP A1 protein is already known to be expressed only in spermatogonia and Sertoli cells (23). Western blotting showed an even more dramatic decrease in protein expression levels in purified cell types, with the corresponding hnRNP A1 protein virtually disappearing in purified meiotic cells (Figure 4B). Other detected meiotic changes in the expression of RNA splicing regulators included activation of each of the genes encoding CUG-binding proteins: *Celf4-6* mRNAs were upregulated over 2-fold during meiosis (Figure 4A and Supplementary Files S5 and S6), and there was also an almost 2-fold upregulation of the *Cugbp1* (*Celf1*) and *Cugbp2* (*Celf2*) genes (Supplementary Files S5 and S6). The expression levels of both *Mbnl1* and *Mbnl2* encoding muscleblind proteins [Mbnl1 interacts with PTB (48)] were downregulated over meiosis (Supplementary Files S5 and S6). Not all changes in mRNA levels resulted in changes in protein expression. Although RNAseq indicated increased or decreased expression of the various *Hnrnph* genes at the transcript level (Supplementary Files S5 and S6), no overall change in expression of the family was seen at the protein level (Figure 4B).

The expression of *Tra2b* mRNA (which encodes the SR-like protein Tra2β) was upregulated almost 2-fold during meiosis (Supplementary Files S5 and S6). In contrast, the expression levels of the classical SR proteins ASF/SF2 (SRSF1), SC35 (SRSF2), SRp40 (SRSF5) and SRp20 (SRSF3) remained similar at both RNA and protein levels between the pre-meiotic and meiotic testis (Figure 4C and Supplementary Files S5 and S6), but subsequently there was a dramatic loss of expression of ASF/SF2, SRp20 and SRp40 during the haploid stages of differentiation. Strong increases in expression during meiosis (∼11- and 2.5-fold, respectively) were observed for the *Sfrs14* mRNA (also known as *Sugp2*), which encodes a relatively uncharacterized SR protein, and for *Sfrs15*, which encodes an SR-like protein (Sca4) that couples transcription and RNA splicing. Expression levels from the *Srpk1* and *Srpk2* genes, which encode serine kinases that phosphorylate SR proteins (and also protamines) (49), also increased between the 6 and 21 dpp testis transcriptomes.

### Specific RNA sequences are associated with meiotically regulated exons in the mouse

**Figure 5.**
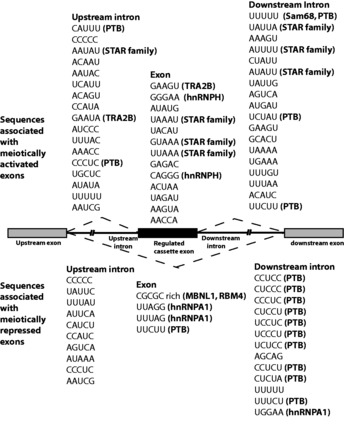
Specific RNA sequences are associated with meiotically regulated exons in the mouse. Frequently occurring 5mers found in and around meiotically regulated exons are shown. In some cases, the RNA binding proteins that might interact with these motifs are indicated. Full details of identified 5mers and their statistical significance are given in Supplementary Table S1.

**Figure 6.**
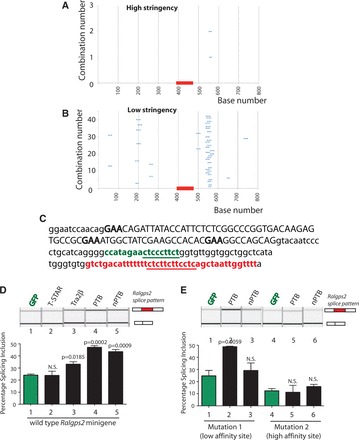
Dissection of the meiotically regulated *Ralgps2* cassette exon. The *Ralgps2* exon and its flanking intron sequences were screened for (**A**) high affinity and (**B**) lower affinity PTB binding sites (the position of the regulated exon is shown on the *x*-axis as a red rectangle). (**C**) Sequence of the meiotically regulated *Ralgps2* exon (upper case) and its flanking intron sequence (lower case). Candidate binding sites for Tra2β in the regulated exons are shown in bold. The intronic PTB binding sites are shown in green (low affinity site, with core motif underlined) and red (high affinity site, with core motif underlined). (**D**) Splicing pattern of transcripts made from a *Ralgps2* minigene in HEK293 cells after co-transfection of expression vectors for different proteins. (**E**) Affect on splicing pattern of transcripts made from the *Ralgps2* minigene after mutation of the low or high affinity PTB binding sites. In parts (D) and (E), the top panel shows a capillary gel electrophoresis analysis from a single experiment, and the bottom panel is a bar chart representing data from three biological replicates.

To unravel the potential roles of changes in RNA protein gene expression in coordinating changes in meiotic splicing profiles, we identified 5mer motifs that were significantly enriched in and around the meiotically regulated cassette exons (Figure 5 and Supplementary Table S1. The statistical significance of enriched 5mers is included in Supplementary Table S1). Identified motifs included known binding sites for PTB (50,51). PTB binding sites were enriched downstream both of activated and repressed exons, similar to the pattern observed downstream of exons positively and negatively regulated in muscle cells (6,52). Binding motifs for PTB upstream of or within an exon are associated with repression by PTB, whereas downstream motifs or motifs close to the splice sites of the adjacent constitutive exon are associated with activation (53,54). Intriguingly, an analysis of the potential binding sites for PTB (38) around the regulated exon in *Ralgps2* suggested that the highest affinity binding sites were downstream of the exon (Figure 6A–C), even though it was activated during meiosis when PTBP1 levels fell (Figure 2).

As germ cells are difficult to transfect *in vitro*, we tested whether the expression of this *Ralgps2* exon might be regulated by PTB using a cell line model. We cloned the regulated exon and its flanking intron sequences into an exon trap vector. Co-transfection of this *Ralgps2* minigene into cells with GFP resulted in production of mainly the exon skipped isoform (Figure 6D, lane 1). However, co-transfection of either PTBP1 or nPTB with the minigene dramatically increased splicing inclusion of the meiosis-regulated *Ralgps2* exon (Figure 6D, compare lane 1 with lanes 4 and 5), as would be expected if PTB bound to the downstream sites.

Our analysis of PTB-binding possibilities, which is based on the sequence preferences of the RNA-binding domains, inter-domain spacing and the number of possible arrangements of binding (38), identified two regions downstream of the *Ralgps2* exon to which PTB might bind; of these, the one to the 3′ side appeared to be much more favourable (Figure 6A–C). To test the individual functions of these sites, they were mutated by converting cytosines in the core CT-rich motif into adenosines (the sequences mutated are underlined in Figure 6C). Mutation of the lower affinity site did not block splicing activation by PTBP1 (lanes 1–3 in Figure 6E), but interestingly it did prevent splicing activation by the nPTB protein, suggesting a slightly different requirement for splicing regulation of this exon by these two highly homologous RBPs. On the other hand, mutation of the higher affinity site prevented splicing activation on co-expression with either PTBP1 or nPTB (compare lanes 4–6 in Figure 6E). We conclude that the exon in *Ralgps2* that is activated in meiosis can be regulated by both PTBP1 and nPTB, both of which act via downstream binding sites to cause inclusion. Although the aforementioned data comes from a reconstituted cell line system, it is suggestive for a potential role for PTB in regulating this *Ralgps2* exon in mouse germ cells. We speculate further that the general enrichment of pyrimidine-rich sequences around the regulated exons is consistent with roles for PTBP1 and nPTB in the regulation of splicing in meiosis.

Other statistically significant motifs shown in Figure 5 associated with inclusion are (G + A)-rich sequences within the exon and UAAAA and similar motifs to the downstream side. These motifs are likely to include binding sites for Tra2β (GAA core site) (55,56) and for Sam68 (57,58) and T-STAR (59,60), which are each highly expressed in testis and upregulated in meiosis (Figure 4 and Supplementary Files S5 and S6). As both *Tra2b* and *Khdrbs1* gene expression changes just <2-fold over meiosis, they are not annotated on Figure 4, although a predicted binding site for Tra2β was the most significant of the recovered 5mers shown in Supplementary Table S1. We tested whether the GAA motif might indicate regulation by Tra2 β using the meiotically regulated exon of *Ralgps2*, which has been shown by CLIP to bind Tra2 β [(27) and data not shown] and contains three GAA motifs (Figure 6C). Co-transfection of a *Ralgps2* minigene with Tra2β caused a small, but statistically significant, increase in inclusion of the *Ralgps2* cassette exon, whereas T-STAR had no effect (Figure 6D, lanes 1–3).

**Figure 7.**
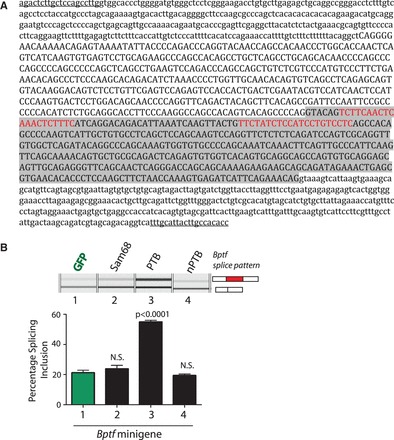
Dissection of the meiotically regulated *Bptf* cassette exon. (**A**) The *Bptf* exon and its flanking intron sequences were screened for high affinity and lower affinity PTB binding sites as in Figure 6. The sequences included as a result of the alternative 5′ site selection are shaded grey, and the high affinity PTB sites are shown in red, just downstream of the alternative 5′ splice site. Exon sequence is shown in upper case, and intron sequence is in lower case. The positions of the cloning oligonucleotides used to make the minigene are underlined. (**B**) Splicing pattern of transcripts made from a *Bptf* minigene in HEK293 cells after co-transfection of expression vectors for different proteins.

We also investigated whether modulations in PTB concentration might regulate other types of high amplitude splicing events, which change over male meiosis. Candidate PTB binding sites (38) were also identified just downstream of the upstream meiosis-regulated 5′ splice site in the *Bptf* gene (Figure 7A). To enable us to test the function of these PTB binding sites on selection of the upstream and downstream *Bptf* 5′ splice sites, we cloned a minigene containing the meiosis-regulated *Bptf* exon with both available 5′ splice sites between β globin exons. When this *Bptf* minigene was co-transfected in HEK293 cells with GFP, we observed mainly use of the upstream 5′ splice site (Figure 7B, lane 1. This is the splicing pattern seen in the mitotically active cells of the testis). In contrast, co-transfection with PTBP1 (but not nPTB) strongly activated use of the downstream 5′ splice site (Figure 7B, lanes 2 and 3. This is the splicing pattern seen in post-meiotic cells in the testis). Splicing control of *Bptf* 5′splice site selection was specific to PTBP1 in these experiments, and no effect on *Bptf* splicing regulation was seen following Sam68 co-transfection.

## DISCUSSION

Here, we have used RNAseq to identify global changes in alternative exon splicing inclusion and parallel switches in the RNA splicing environment during mouse male meiosis. Our data reveal that quantitatively significant protein-coding splicing changes occur during mouse male meiosis. The work described here builds on previous work that detected extremely high levels of overall alternative splicing in the whole testis, but which concluded that much of this is likely to be non-functional based on the low amplitude of the changes, poor conservation and low protein-coding potential (13). In contrast, the meiotically regulated switches we describe here have high fold changes and are also regulated at some frequency in other tissues. For example, the meiosis-selected *Bptf* alternative 5′ splice site is also selected in the heart and muscle as well as the testis. Exon skipping was the most frequently identified form of alternative splicing regulation between the 6 and 21 dpp testis transcriptomes (Figure 1B), and exon skipping is also the highest frequency alternative splice event in the mouse transcriptome (61).

Most exons are under combinatorial control from different splicing regulator proteins and also contributions from transcription-related effects (44,45). Although the cassette exon splice switches in the *Odf2* and *Nasp* genes were also associated with the concurrent use of alternative promoters in meiosis, our data suggest that global changes in the concentration of RNA splicing regulators during meiosis make important contributions to the observed switches in splicing. One striking change is a switch between *Ptbp1* and *Ptbp2* gene expression in meiosis. A similar switch is seen in neurogenesis (62–64). Both the encoded PTB proteins (PTBP1 and nPTB) are generally seen as repressors of splicing (65,66), although it is not clear whether nPTB is a weaker repressor than PTBP1 as originally suggested (64,65,67). Both PTB proteins are also able to activate splicing, although the dependence of activation versus inhibition of an exon on the location of the PTB binding sites is not clear (53,54). In HeLa cells, it appears that the two proteins affect the same targets (53,68), whereas in neuroblastoma cells, the proteins also affect separate sets of exons (62). It is, therefore, difficult to predict whether the switch from PTBP1 to nPTB would contribute to the observed splicing changes accompanying meiosis. In the case of the meiotic exon of *Ralgps2*, both PTBP1 and nPTB proteins increased inclusion in transfected cells using minigene constructs (Figure 6). Direct investigation of the regulation of these exons *in situ* will require the utilization of appropriate mouse knockout models (germ cells are not easily transfected *in vitro*). Interestingly, though, whereas both PTBP1 and nPTB depended on the presence of a good candidate downstream binding site for their splicing effect, nPTB also required a further weaker site that would not have been detected by the common practice of searching for sequences containing UCUU or (CU)_n_. It would be interesting to know whether the presence of such additional motifs is a characteristic of exons regulated by nPTB.

Other regulatory proteins that might be important in activating meiotic splicing of the *Ralgps2* exon include Tra2β. The *Tra2b* gene was also upregulated in meiosis, and the *Ralgps2* exon contained GAA target motifs and was activated by Tra2β. The *Ralgps2* exon was also identified as a Tra2β-CLIP tag in mouse testis (AJ Best and DJ Elliott, data not shown). Other exons identified by RNAseq here that are known from CLIP analysis in the mouse testis to be bound strongly *in vivo* by Tra2β are the cassette exon of *Nasp-T* and poison exon of *Tra2b* (27,69).

Our transcriptome-wide analysis also identified changes affecting the expression of other proteins that regulate splicing. These include the replacement of RBMX with RBMXL2 (26) and the meiotic upregulation of T-STAR and Sam68 (28,29,60). Predicted target sites for Sam68 and T-STAR splicing regulators were enriched downstream of activated exons, and Sam68 protein is known to regulate a cassette exon in the *Sgce* gene in meiosis that has a downstream UAAA-rich site (70). Expression of these RNA-binding proteins is known to be important for male germ cell development. Haploinsufficiency of *Rbmxl2* causes infertility in mice (47), and Sam68 null mice are infertile (71,72). A number of unanticipated changes were also found in splicing regulator gene expression. Members of the CELF protein group, including CUG-BP2, were upregulated in meiosis. This change is likely to be important as the *Celf1* gene encoding CUG-BP1 is essential for spermatogenesis in mice (73). CELF proteins often work in antagonism to the muscleblind proteins (74), which were themselves transcriptionally repressed during meiosis. Target binding sites for CUG-BP2 and muscleblind proteins were also respectively enriched within activated and repressed exons (Figure 5).

Previous data have shown that the transcription of a core panel of genes changes during meiosis and provides many of the structural components needed for this unique division cycle (18–20). Many of the genes affected are expressed only in the testis (e.g. the genes encoding synaptonemal complex proteins) (18). In contrast, many of the exons identified here as being under meiotic splicing control are included to some extent in other mouse tissues. However, two of the substantial switches in splicing patterns identified here by RNAseq have already been associated with important roles in animal germ cell development. Meiotic skipping of the *Odf2* exon is associated with a switch in protein function from a somatic intracellular role in organising microtubules within the centriole to a post-meiotic role in organizing microtubules in the sperm tail (75,76). Alternative splicing of the *Nasp* gene creates a protein isoform associated with meiotic chromosomes that forms part of the machinery that monitors DNA integrity during meiosis (77–79). Quantitative meiotic splicing regulation also takes place in other genes implicated in key roles in germ cell development. The *Ezh2* gene encodes an important chromatin modifier that can affect development (80) and might play an important role in normal fertility (81,82). A mutually exclusive exon is selected in the *Ate1* gene, and the meiotic *Ate1* mRNA isoform is the major mRNA made from this gene in the mouse testis (Supplementary File S1). Knockout of the *Ate1* gene prevents germ cell development in the mouse (83). The major switches in alternative splicing events discovered here might thus underlie essential changes in the expression of meiotic protein isoforms that play significant roles in preparing the cell for the morphological transformations that lie ahead.

## SUPPLEMENTARY DATA

Supplementary Data are available at NAR Online.

## FUNDING

Wellcome Trust [WT080368MA and WT089225/Z/09/Z to D.J.E.]; BBSRC [BB/D013917/1 and BB/I006923/1 to D.J.E.]; Telethon Grant [GGPGGP09154]; Associazione Italiana Ricerca sul Cancro (AIRC) 2010 (to C.S.); Addison Wheeler trust (to S.G.). Funding for open access: Wellcome Trust.

*Conflict of interest statement*. None declared.

## Supplementary Material

Supplementary Data
