# Supplementary material for: The splicing landscape is globally reprogrammed during male meiosis
Source: Nucleic Acids Res. 2013 Sep 12;41(22):10170–84. doi: 10.1093/nar/gkt811 (PMC3905889; doi:10.1093/nar/gkt811)
Supplement: Supplementary Data [file supp_41_22_10170__index.html]

The splicing landscape is globally reprogrammed during male meiosis — The splicing landscape is globally reprogrammed during male meiosis — Supplementary Data 

# The splicing landscape is globally reprogrammed during male meiosis

## Supplementary Data

files

**Files in this Data Supplement:**

- Supplementary Data - pdf file
- Supplementary Data - xls file
