# Supplementary material for: The splicing landscape is globally reprogrammed during male meiosis
Source: Nucleic Acids Res. 2013 Sep 12;41(22):10170–84. doi: 10.1093/nar/gkt811 (PMC3905889; doi:10.1093/nar/gkt811)
Supplement: Supplementary Data [file supp_gkt811_nar-02031-a-2013-File009.pdf]

Supplementary Material Legends

Supplementary Figure 1. Genes regulated at the level of alternative splicing control during meiosis can also be transcriptionally regulated. (A) Scatterplot analysis of a panel of genes that contain meiotically regulated alternative exons, showing relative expression levels in the day 6 versus day 20 transcriptomes. Genes that contain exons activated in meiosis are shown as red dots, and genes that contain exons that are skipped in meiosis are shown as blue dots The Legend for the numbers is given in Supplementary File 4. (B) Table summarising the numbers of transcriptionally up-regulated and down-regulated genes (defined as a more than 2-fold change in gene expression) which showed either increased or decreased splicing inclusion of cassette exons during meiosis. (C), (D). Meiotic splicing regulation of the *Odf2* mRNA and *Nasp* mRNAs respectively is associated with selection of additional promoters during meiosis.

Supplementary Figure 2. RNAseq analysis of overall gene expression changes in meiosis. Scatterplot showing relative gene expression profiles the mitotic (6 dpp) and postmeiotic (21 dpp) testes. Each dot on the scatterplot represents the relative gene expression profile of an individual gene at 6 dpp and 21 dpp, plotted on logarithmic scales. Genes above the solid diagonal line are expressed more highly at 21 dpp, and genes below the diagonal line are more highly expressed at 6 dpp. The broken diagonal line indicates the position of a 2-fold up or down-regulation of transcript levels between 6 dpp and 21 dpp transcriptomes. The position of known genes from the reported core mitotic or meiotic transcriptomes [14] are indicated in red. Individual gene numbers correspond to: 1:*4930550C14Rik*, 2:*Abca1*, 3:*Acrbp*, 94:*Adam18*, 5:*Adam2*, 6:*Als2cr11*, 7:*Aurkc*, 8:C*age1*, 9:*Ccdc65*, 10:*Ccdc67*, 11:*Ccna1*, 12:*Dmrt1*, 13:*Gata4*, 14:*Gsto2*, 15:*Ldhal6b*, 16:*Lrrc27*, 17:*March10*, 18:*March11*, 19:*Nasp*, 20:*Nmnat3*, 21:*Osr2*, 22:*Pcdh18*, 23:*Pih1d2*, 24:*Pla2g6*, 25:*Ppp3r2*, 26:*Smcp*, 27:*Spag6*, 28:*Spo11*, 29:*Sycp2*, 30:*Tcfl5*, 31:*Tdrd1*, 32:*Tekt5*, 33:T*np1*, 34:*Ypel1*, 35:*Zpbp*.

Supplementary Table 1 K-mer enrichment analysis of cassette exons differentially spliced in meiosis and flanking 250bp of intron sequence. Each group of K-mers are ordered according to significance of p value. (A) Exons activated in meiosis. (B) Exons repressed in meiosis. Parts (A) and (B) are split up into (i) exons; (ii) upstream introns; and (iii) downstream introns.

Supplementary File 1.
Sequences of the oligonucleotides used in this study.

Supplementary File 2 Predicted mouse meiotic alternative splicing events.

Supplementary File 3 Gene expression levels of genes containing meiotically regulated exons.

Supplementary File 4 Core changes between mitotic and meiotic transcriptome. The expression profiles (RNAseq reads/gene) in the 6 dpp and 21 dpp testis transcriptomes are given for members of the core mitotic and meiotic transcriptomes (17). The numbers given for each gene are the same as in Supplementary Figure 2A, so this file provides a legend for this scatterplot.

Supplementary File 5. The expression pattern of genes encoding all known RNA splicing regulators in the 6 dpp and 21 dpp testis transcriptomes monitored by RNAseq.

Supplementary File 6. RNA binding proteins which show biggest changes in gene expression over meiosis, along with their predicted binding sites where known. Downregulated genes are shown in blue text, up-regulated in black text.

(A)

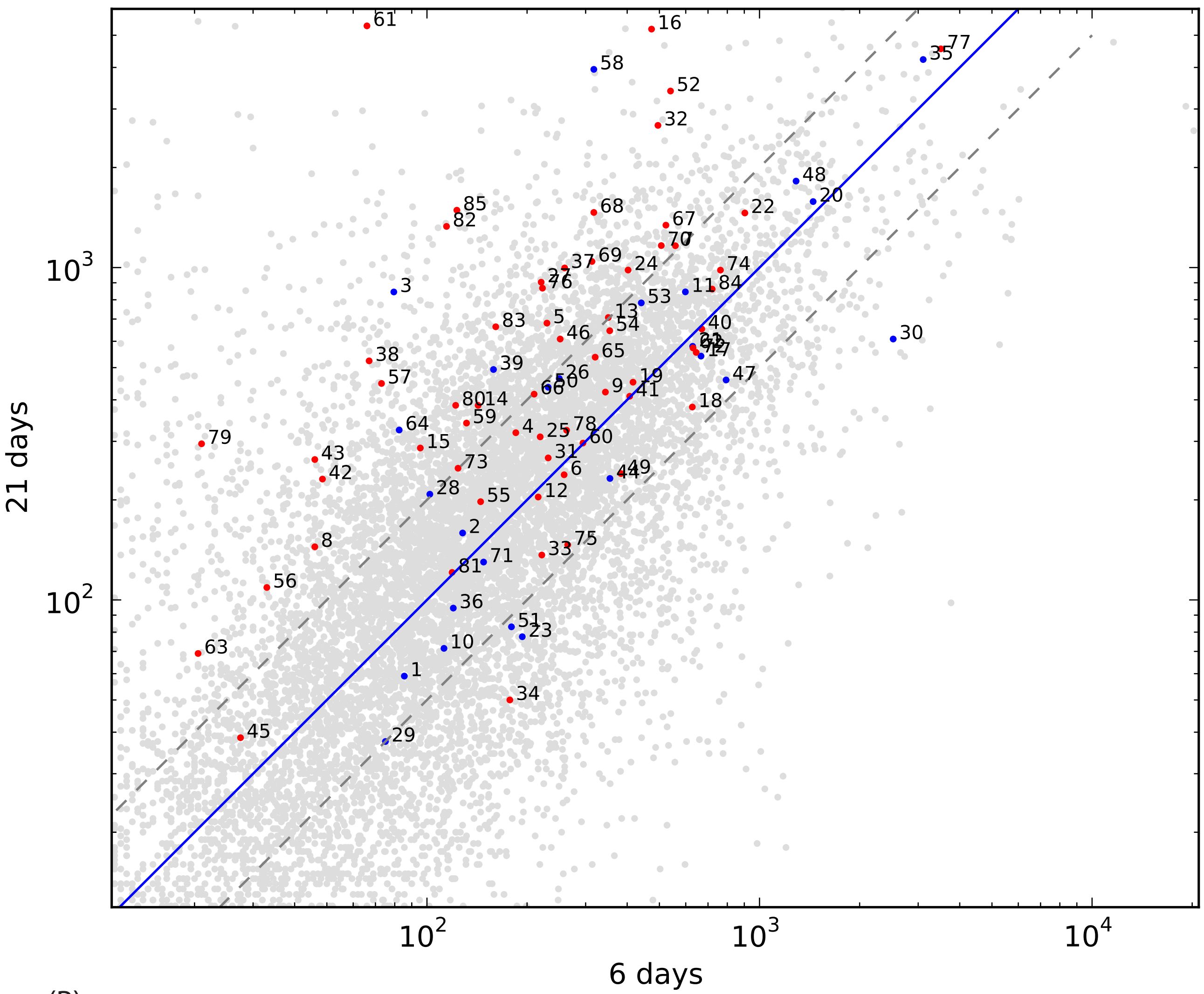

(B)

Transcriptionally activated genes	Transcriptionally repressed genes
30 meiotically up-regulated genes showed increased splicing inclusion of a cassette exon	1 meiotically down-regulated gene showed increased splicing inclusion of a cassette exon.
5 meiotically up-regulated genes showed increased exon skipping of a cassette exon	3 meiotically down-regulated genes showed increased exon skipping of a cassette exon

(C) (D)

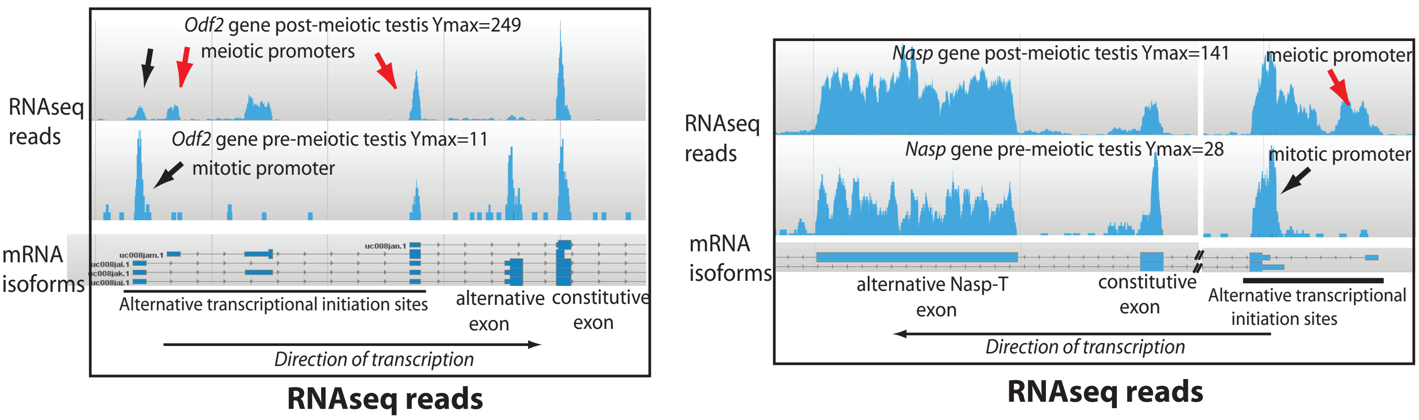

Elliott, Supplementary Figure 1

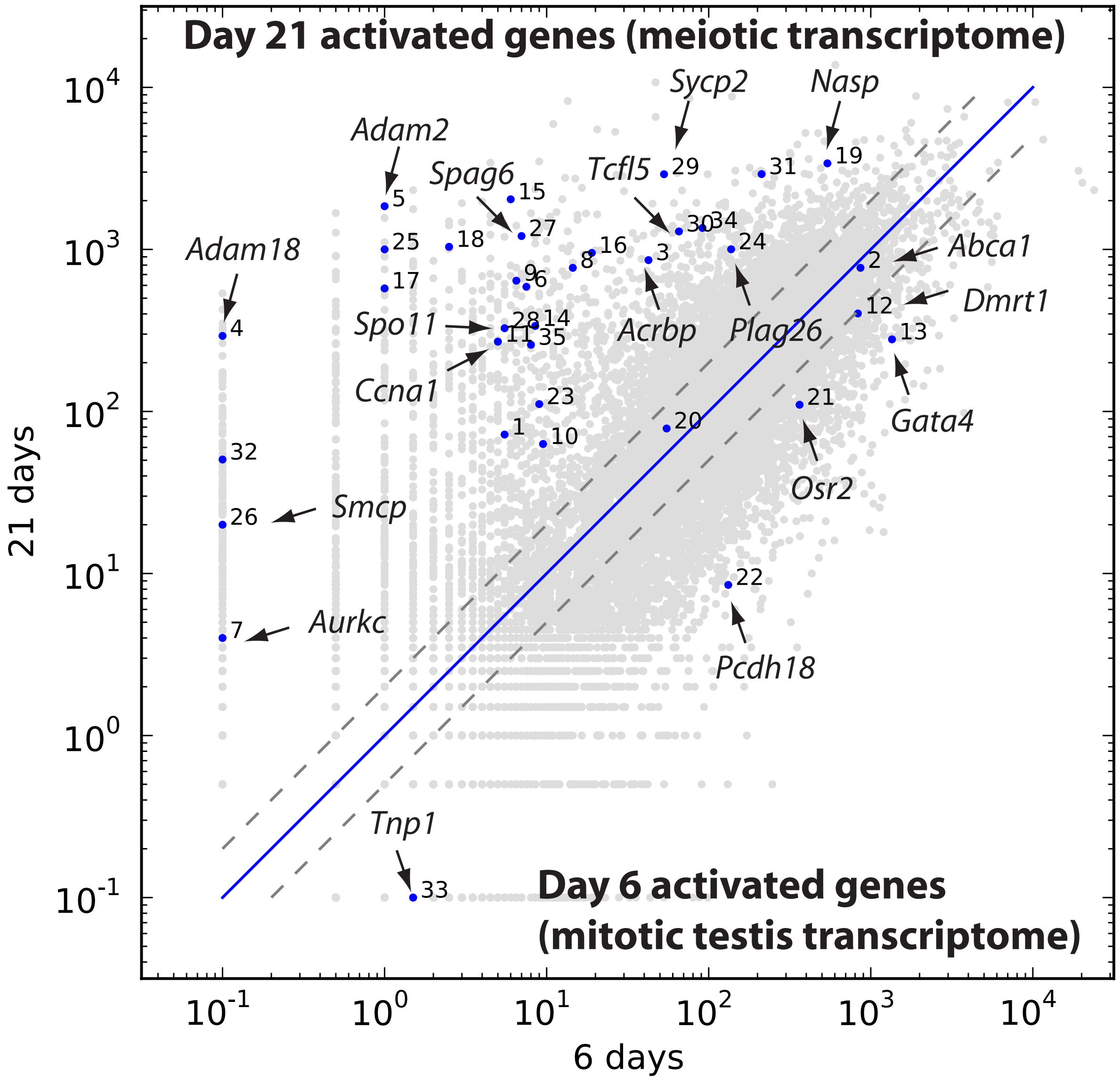

Elliott et al, Supplementary Figure 2

Supplementary Table 1
K-mer enrichment analysis of exons and flanking 250b introns differentially spliced in meiosis

(A) Exons activated in Meiosis:

i) Exons

k-mer	Normalised counts in meiosis activated exons	Normalised counts in background exons	Background corrected k-mer occurrence in Meiosis activated exons	p value	Known splicing factor binding site
GAAGT	111	52	59	0.001526	Tra2β
GGGAA	106	51	55	0.002656	hnRNPh
ATATG	50	17	33	0.006691	Rbmxl2, KhdRbs3
TAAAT	45	14	31	0.007317	Rbmxl2, KhdRbs3
TACAT	61	26	35	0.011002	
GTAAA	50	19	31	0.011364	Rbmxl2, KhdRbs3
TTAAA	63	28	35	0.011430	Rbmxl2, KhdRbs3
GAGAC	84	44	40	0.013297	
CAGGG	106	60	46	0.013299	hnRNPh
ACTAA	40	13	27	0.014178	
TAGAT	42	15	27	0.015478	
AAGTA	61	28	33	0.017159	
AACCA	74	38	36	0.018720	
AATAA	61	29	32	0.021208	Rbmxl2, KhdRbs3
TTTGT	69	35	34	0.022226	
GGAAT	53	24	29	0.024321	Tra2β
CTAAC	40	15	25	0.024504	
CGTTT	32	10	22	0.025102	
AATCA	63	32	31	0.026399	
ATTTG	71	38	33	0.027002	
TGTAT	53	25	28	0.030225	
TTTAA	58	29	29	0.031366	Rbmxl2, KhdRbs3
GGCAG	161	112	49	0.034560	

TTAGT	45	20	25	0.034578	
TATCA	47	22	25	0.035676	
ATTAG	34	13	21	0.036275	
TTTTA	53	26	27	0.037271	Rbmxl2, KhdRbs3
TTGAG	79	46	33	0.037786	
AGTTG	61	32	29	0.038471	
AATTT	71	40	31	0.038515	
GTCCG	37	15	22	0.038555	
TTAGG	21	5	16	0.038671	
TGCGT	21	5	16	0.038671	
TTCTT	92	57	35	0.041459	PTB
CTTAA	42	19	23	0.042020	
ACTTA	45	21	24	0.043283	
GATCG	32	12	20	0.044253	
TTTCA	71	41	30	0.045638	
GAGTG	71	41	30	0.045638	
TTGGG	61	33	28	0.046317	
TCCGG	37	16	21	0.049310	

ii) Upstream introns

k-mer	Normalised counts in meiosis activated upstream introns	Normalised counts in background upstream introns	Background corrected k-mer occurrence in Meiosis activated upstream introns	p value	Known splicing factor binding site
CATTT	201	120	81	0.000174	YBX1
CCCCC	108	55	53	0.000715	
AATAT	122	66	56	0.000851	Rbmxl2, KhdRbs3
ACAAT	68	33	35	0.004681	
AATAC	80	43	37	0.006251	
TCATT	149	98	51	0.006655	
ACAGT	106	64	42	0.007795	
CCATA	62	32	30	0.010096	
GAATA	80	46	34	0.012730	SFRS10
ATCCC	83	49	34	0.013648	

TTTAC	115	74	41	0.014060	
AAACC	75	43	32	0.016012	
CCCTC	137	94	43	0.017933	
TGCTC	125	84	41	0.018163	
ATATA	108	70	38	0.018893	
TTTTT	625	531	94	0.019484	
AATCG	14	2	12	0.019995	
ATCGC	16	3	13	0.021449	
CATAT	73	43	30	0.021639	
CGCAA	17	4	13	0.022225	
CAGTT	108	71	37	0.022560	
TTTCC	189	140	49	0.022994	
ATATG	85	53	32	0.023632	
GTTTC	142	101	41	0.026709	
CATAG	64	38	26	0.031951	
TACCT	87	56	31	0.032196	
CATCT	118	83	35	0.038926	
TTCTT	240	189	51	0.039848	PTB
ACATT	128	92	36	0.039981	
TCCCC	144	106	38	0.043622	
TGCAT	111	78	33	0.044336	
CCCCA	128	93	35	0.046105	
TATTT	200	155	45	0.046311	Rbmxl2, KhdRbs3
ATTAT	106	74	32	0.047042	
TATCC	56	33	23	0.047489	
TATAG	82	54	28	0.048582	

iii) Downstream introns

k-mer	Normalised counts in meiosis activated downstream introns	Normalised counts in background udownstream introns	Background corrected k-mer occurrence in Meiosis activated downstream introns	p value	Known splicing factor binding site
TTTTT	540	356	184	0.000000318	Sam68
TATTA	109	56	53	0.000666282	Rbmxl2, KhdRbs3
AAAGT	120	66	54	0.001157977	

ATTTT	248	170	78	0.001417088	Rbmxl2, KhdRbs3
CTATT	90	48	42	0.003136229	
ATATT	109	63	46	0.003551768	Rbmxl2, KhdRbs3
TATTG	89	48	41	0.004319700	
AGTCA	94	54	40	0.006950865	
ATGAT	95	56	39	0.008031022	
TCTAT	92	54	38	0.009338319	PTB
GAAGT	89	52	37	0.010864321	SFRS10
GCACT	83	48	35	0.010933258	
TAAAA	168	117	51	0.011099812	Rbmxl2, KhdRbs3
TGAAA	132	87	45	0.011499098	SFRS10
TTTGT	236	176	60	0.013061238	
TTTAA	220	164	56	0.015759749	Rbmxl2, KhdRbs3, Sam68
ACATC	62	34	28	0.016993155	
TTCTT	210	156	54	0.017868926	PTB
TCAAA	95	60	35	0.018228399	
TGTAT	127	86	41	0.019925583	
TAACA	89	55	34	0.020287395	
TTATA	101	65	36	0.021212276	
TTTTG	201	150	51	0.021569030	
CAACA	76	46	30	0.023017214	
TGTTT	241	186	55	0.024585328	
ATCTA	69	41	28	0.025271899	
GTTAG	85	54	31	0.028740641	
ATCCA	75	46	29	0.030651279	
ATTAT	95	63	32	0.031788968	
TAAGC	78	49	29	0.032130969	
GTATT	99	66	33	0.032639149	
AAACG	23	8	15	0.033939659	
TTCAT	125	88	37	0.034201708	
CCTTA	80	51	29	0.036164052	
AATAT	90	60	30	0.039789035	
AATGA	97	66	31	0.041663192	
TATTC	68	42	26	0.041882655	
AAAAT	165	124	41	0.043430741	
GAAAC	80	52	28	0.043680361	
TATCA	71	45	26	0.043822615	
GCCGC	35	17	18	0.043845113	
CATCC	92	62	30	0.043924062	
AAATG	146	108	38	0.046618137	

AGTTA	90	61	29	0.047319495	
ATATC	56	33	23	0.047489372	
ACAAT	50	29	21	0.047922243	

(B) Exons repressed in Meiosis

i) Exons

k-mer	Normalised counts in meiosis repressed exons	Normalised counts in background repressed exons	Background corrected k-mer occurrence in Meiosis repressed exons	p value	Known splicing factor binding site
CGCGC	61	8	53	0.002014	MBNL1, RBM4
TTAGG	47	5	42	0.005230	hnRNPA1
CGCCG	56	12	44	0.007733	MBNL1, RBM4
GCGGC	70	21	49	0.008508	MBNL1, RBM4
CGGCC	66	19	47	0.009949	MBNL1, RBM4
CCGCG	47	8	39	0.010027	MBNL1, RBM4
TTTAG	70	23	47	0.011834	hnRNPA1
CCCGG	84	33	51	0.013193	MBNL1
TTCTT	117	57	60	0.014703	PTB
GCGCG	37	5	32	0.015679	
GCGCC	61	19	42	0.016376	
CGGCG	47	11	36	0.018264	
AAGTG	117	60	57	0.020785	
CCATG	112	57	55	0.021953	
GCCGC	66	24	42	0.022428	
GCCCG	66	25	41	0.026104	
AACCA	84	38	46	0.026175	
GTAAA	56	19	37	0.026885	
CGCCC	56	19	37	0.026885	
TGCCT	136	78	58	0.030722	
ACCAT	84	40	44	0.033796	
CCGGC	66	27	39	0.034990	
CGTAA	28	4	24	0.038530	
ATTTA	66	28	38	0.040301	

CAAGT	103	56	47	0.042943	
GATTA	51	19	32	0.043980	

ii) Upstream introns

k-mer	Normalised counts in meiosis repressed upstream introns	Normalised counts in background repressed upstream introns	Background corrected k-mer occurrence in Meiosis repressed upstream introns	p value	Known splicing factor binding site
CCCCC	117	55	62	0.002092	
TATTC	102	49	53	0.004910	
TTTAT	240	160	80	0.007002	STAR family
ATTCA	123	67	56	0.007322	
CATCT	141	83	58	0.009927	
CCATC	114	63	51	0.011272	
AGTCA	108	60	48	0.014308	
ATAAA	135	82	53	0.016323	
CCCTC	150	94	56	0.016353	
AATCG	21	2	19	0.018442	
CTTGG	129	79	50	0.020549	
ATTTT	273	200	73	0.022207	
AAACC	81	43	38	0.024643	
TCATT	150	98	52	0.026331	
AAAAA	267	197	70	0.026707	
AGAGT	102	60	42	0.028139	
TCGTG	36	12	24	0.028462	
CCTCC	156	104	52	0.029691	
GTTTA	114	70	44	0.030182	
ATTTA	177	122	55	0.031375	
CAATT	90	52	38	0.034207	
CTGAA	126	81	45	0.035695	
TTATT	192	136	56	0.035847	
GTTCA	99	60	39	0.039011	
TCTAT	90	53	37	0.039522	
AGGAC	90	53	37	0.039522	
CTCAA	99	61	38	0.044608	
CTCTA	105	66	39	0.045716	
ATTCC	81	47	34	0.045877	

ATCGC	18	3	15	0.046948	
GCATT	96	59	37	0.047007	
GAGTC	78	45	33	0.048197	
TATTT	210	155	55	0.049645	

iii) Downstream introns

k-mer	Normalised counts in meiosis repressed downstream introns	Normalised counts in background repressed downstream introns	Background corrected k-mer occurrence in Meiosis repressed downstream introns	p value	Known splicing factor binding site
CCTCC	264	109	155	0.000000242	PTB
CTCCC	243	109	134	0.000003680	PTB
CCCTC	210	100	110	0.000046795	PTB
CTCCT	183	102	81	0.001500726	PTB
TCCTC	150	79	71	0.002022782	PTB
TCCCT	219	134	85	0.002509716	PTB
TCTCC	153	87	66	0.004735900	PTB
AGCAG	192	118	74	0.004970605	
CCTCT	177	113	64	0.011672221	PTB
CTCTA	108	59	49	0.012289081	PTB
TTTTT	456	356	100	0.016023931	
TTTCT	276	199	77	0.016275066	PTB
TGGAA	153	98	55	0.019785896	hnRNPA1
ATAAC	66	31	35	0.020732288	
ATATT	108	63	45	0.022170201	STAR family
GCATG	129	82	47	0.029989871	
TCAAC	60	29	31	0.032010323	
ACCCG	27	7	20	0.033008942	
GCAAT	66	34	32	0.035652246	
GGAAA	126	81	45	0.035694659	
TAAAG	129	84	45	0.038129471	
CATAA	72	39	33	0.038780341	
AGGGA	144	97	47	0.041008006	
